# A Convenient Chloroform Package

**Published:** 1909-10

**Authors:** 


A Convenient Chloroform Package.—Much interest is being manifested in the chloroform dropper-ampoule marketed by Parke, Davis & Co., and which, in the opinion of a good many physicians and surgeons, is the most convenient and practical chloroform package that has ever been introduced to the profession. The new device is at once a hermetically sealed container and a perfect dropping bottle that can be carried about in the emergency bag at all times in readiness for immediate use. It supplies in portable form enough of the anesthetic for one service—about thirty grammes. The desirability of such an individual package and its superiority over the ordinary amber, cork-stoppered bottle heretofore supplied is appreciated when one remembers that chloroform in broken packages rapidly deteriorates under the influence of air and light and becomes contaminated with chlorine decomposition products.

The dropper-ampoule is, furthermore, a very economical package, as loss by evaporation, spilling of contents, and deterioration are practically eliminated. The chloroform may be dropped directly upon the mask with ease and accuracy. The anesthetist has perfect control of the outflow and is enabled to regulate at his discretion the intervals between drops.

Physicians desiring further information relative to the dropper-ampoule are advised to write to Parke, Davis & Co. for their illustrated circular descriptive of the new package, addressing them either at their main laboratories, Detroit, Mich., or any of their branches.

